# Mortality following cancer diagnosis among people with non-affective psychoses: mediation by stage at diagnosis and time to treatment initiation

**DOI:** 10.1007/s00127-026-03063-x

**Published:** 2026-03-05

**Authors:** Jared C. Wootten, Lucie Richard, Igor Karp, Phillip S. Blanchette, Marco Solmi, Kelly K. Anderson

**Affiliations:** 1 Department of Epidemiology and Biostatistics, Schulich School of Medicine and Dentistry, Centre for Public Health and Family Medicine, Western University, Room 3146A 1465 Richmond Street, N6G 2M1 London, ON Canada; 2 Psychiatry, Western University, London, ON Canada; 3 ICES Western, London, ON Canada; 4 Division of Medical Oncology, Department of Oncology, Verspeeten Family Cancer Centre, London Health Sciences Centre, Western University, London, ON Canada; 5 Department of Psychiatry, University of Ottawa, Ottawa, ON Canada; 6 Regional Centre for the Treatment of Eating Disorders and On Track, The Champlain First Episode Psychosis Program, Department of Mental Health, The Ottawa Hospital, Ottawa, ON Canada; 7 Ottawa Hospital Research Institute (OHRI) Clinical Epidemiology Program, University of Ottawa, Ottawa, ON Canada; 8 Department of Child and Adolescent Psychiatry, Charité Universitätsmedizin, Berlin, Germany

**Keywords:** Psychosis, Schizophrenia, Cancer, Mortality, Stage at diagnosis, Treatment, Mediation

## Abstract

**Background:**

People with psychotic disorders have greater mortality rates following cancer diagnosis, compared to people without psychotic disorders. Prior examinations of the effect of psychotic disorders on survival following cancer diagnosis mediated by stage at diagnosis and treatment disparities did not accommodate for multiple mediators and post-exposure confounding. The present study sought to estimate analogues of the natural indirect effects of having NAPD on mortality following cancer diagnosis, mediated through stage at diagnosis and time to treatment initiation.

**Methods:**

We identified cases of cancer diagnosed between 1995 and 2019 among people with non-affective psychotic disorders (NAPD) and a comparison group without NAPD, constructed using Ontario health administrative data. Death from any cause was identified using register data. Inverse probability of treatment weighted Cox models were used to estimate the effect of NAPD on mortality following cancer diagnosis mediated by stage at diagnosis and time to treatment initiation, adjusting for relevant confounders.

**Results:**

The analytic sample included 3,643 people with NAPD and 15,174 people without NAPD who developed cancer. People with NAPD had a 66% greater adjusted hazard of all-cause mortality than people without NAPD (95%CI = 1.55,1.78). The HR estimate for the indirect effect mediated through stage at diagnosis was 1.09 (95%CI = 1.05,1.13) and HR estimate for the indirect effect mediated through time to treatment initiation was 1.00 (95%CI = 0.96,1.04).

**Conclusions:**

Our findings suggest that a relatively small proportion of the effect of NAPD on mortality is mediated by stage at diagnosis, while time to treatment initiation does not mediate that effect. This excess risk is potentially mediated by other patient, provider, and system-related factors in cancer care.

**Supplementary Information:**

The online version contains supplementary material available at 10.1007/s00127-026-03063-x.

## Introduction

 People with psychotic disorders often receive inferior care for physical illnesses and experience greater rates of mortality as a result [1]. In previous studies, we found that people with non-affective psychotic disorders (NAPD), including schizophrenia spectrum disorder, had 23% greater odds of having more advanced stage cancer at diagnosis, were 13% less likely to receive treatment following cancer diagnosis, and had a 68% greater risk of all-cause mortality following cancer diagnosis [2, 3]. Intuitively, it has been suggested that the difference in risk of death following cancer diagnosis between people with psychosis and people without is mediated by differences in stage at diagnosis and the care received [4, 5]. Stage at diagnosis is a crucial diagnostic element, indicating the extent of cancer spread and tumour size. People diagnosed with distant cancer can have 20 times the hazards of mortality compared to those with localized tumours [6]. Timely treatment initiation is also a crucial predictor of mortality following cancer diagnosis, with each 4-week delay in receipt of surgery associated with an estimated 6–8% increase in mortality risk [7]. For those diagnosed with colorectal cancer, each 4-week delay in the receipt of chemotherapy is associated with 13% greater mortality risk [7].

**Fig. 1:**
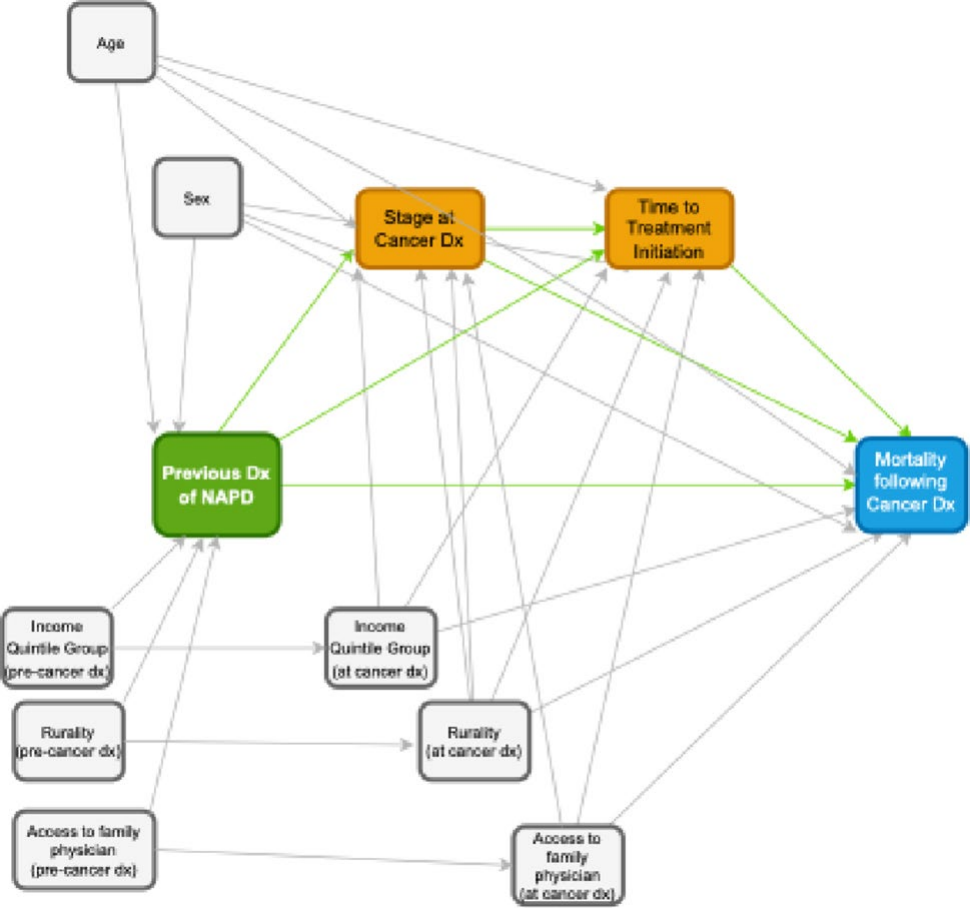
Directed acyclic graph (DAG) illustrating causal relationship between diagnosis with NAPD and all-cause mortality following cancer diagnosis

Prior studies have attempted to decompose the effect of having a diagnosed NAPD on mortality following cancer diagnosis mediated through stage at diagnosis and treatment disparities on cancer mortality among people with NAPD [8–11]. However, these studies did not account for post-exposure confounding by multiple mediators in the causal structure of these variables (Fig. 1). Due to the inherent influence of stage at diagnosis in informing care, stage at diagnosis may act as a post-exposure confounder of the effect mediated through time to treatment initiation, rendering the natural indirect effects through time to treatment initiation unidentifiable when using ‘traditional’ regression analyses and marginal structural models [12].

The aim of the present study was to elucidate the mediating roles of these two factors in the excess mortality risk following cancer diagnosis among people with NAPD. Specifically, we aimed to estimate analogues of the natural indirect effects of having NAPD on mortality following cancer diagnosis, mediated through stage at diagnosis and time to treatment initiation.

## Methods

This manuscript followed the REporting of studies Conducted using Observational Routinely-collected health Data (RECORD) statement [13], available in Supplemental Table 1.

### Data sources

We conducted a retrospective cohort study using population-based health administrative data from Ontario, Canada, held by ICES (formerly the Institute for Clinical Evaluative Sciences). ICES is an independent, non-profit research institute whose legal status under Ontario’s health information privacy law allows it to collect and analyze health care and demographic data, without consent, for health system evaluation and improvement. Healthcare in Ontario is publicly funded, universal, and free at point of use for nearly the entire Ontario population. The data holdings were accessed at ICES, and contained information on hospital admissions, emergency department visits, outpatient physician billings, and data on patient characteristics. Databases were linked at the patient level using unique, encoded identifiers. A description of the databases used in this study can be found in Supplemental Table 2. The use of the data in this project is authorized under Sect.  45 of Ontario’s Personal Health Information Protection Act (PHIPA) and does not require review by a Research Ethics Board.

### Study design

The present analytic sample was selected from a prior retrospective cohort study. The broader cohort included people in Ontario between January 1995 and December 2004 who were born after 1935. We excluded anyone with invalid or missing age and sex variables, non-Ontario residents, people who were ineligible for OHIP in the previous year, those diagnosed with cancer prior to 1995, or anyone with an appearance of a billing code for NAPD from 1990 to 1995. From this group, we then identified people aged 14 to 59 years at the time of diagnosis with NAPD using a validated algorithm (Supplementary Table 1) [14]. Affective psychoses were not included, as they cannot be reliably identified in the outpatient billings. People who did not have evidence of a diagnostic code for NAPD were then randomly sampled and frequency-matched by age and sex. In the current study, we restricted the analytic sample to all people from the broader cohort who had an incident cancer diagnosis between 01-January-2007 and December-30-2019, as identified using the Ontario Cancer Registry (OCR) [15]. Only cancer cases diagnosed on or after 01-January-2007 were included in the analytic sample, as stage at diagnosis was not collected in the OCR prior to this date. We excluded anyone with unknown stage at diagnosis or missing data on neighbourhood-level income, rurality of residence, or access to a family physician at the time of cancer diagnosis (< 1.5%). Given the small amounts of missingness for these variables, complete case analyses were performed, as multiple imputation provides little benefit for missingness < 5% [16]. There were 1447 (39.7%) and 5448 (36.0%) cases with unknown or missing stage at diagnosis among people with NAPD and people without NAPD, respectively. Unknown or missing stage at diagnosis so not reasonable to impute due to the variety of mechanisms of missingness which included unstaged cancers, unrecorded stage at diagnosis, and stage data missing at random from the dataset. The analytic sample was followed until death or one of the following censoring events (whichever happened first): an incident NAPD diagnosis in the comparison group, loss of contact with OHIP (e.g. OHIP ineligibility, leaving Ontario), or the end of the follow-up period (31-December-2019).

## Variables

### Potential confounders

Potential confounding variables were measured at the date of cancer diagnosis. Age and sex were defined as continuous and binary variables, respectively. Neighborhood-level income quintile was assigned based on the census data for each postal code. Rural residences were defined as communities above the 40th percentile in the rurality index of Ontario (RIO). Access to a family physician was defined based on whether each person was assigned to a family physician at the time of cancer diagnosis. Comorbidities at the time of cancer diagnosis were represented by the John Hopkins aggregated diagnostic groups (ADG) algorithm, which used data from prior OHIP billings and hospital discharges to group diagnoses by duration, severity, diagnostic certainty, etiology, and specialty care involvement [17]. A validation study using Ontario health administrative data demonstrated a high discriminatory ability of logistic models including the ADG score in predicting 1-year mortality among people diagnosed with schizophrenia [18]. Cancer site was based on International Classification of Diseases for Oncology (3rd Edition) [19] topography codes for the primary tumor site.

### Proposed mediators

Cancer stage at diagnosis was classified using the Tumour, Node, and Metastases (TNM) American Joint Classification of Cancer Staging criteria for the primary tumour site [20], and represented by an ordinal variable with 4 levels corresponding to stages I through IV. For sensitivity analyses, we created a binary variable, classified as having advanced (stages III/IV) versus local/regional stage cancer at diagnosis (I/II). For another sensitivity analysis, we also created a binary, composite variable, classified as being either: (1) having advanced (stages III/IV)/missing stage at diagnosis; or (2) local/regional stage cancer at diagnosis (I/II).

Radiotherapy, chemotherapy, and oncology surgeries were identified through physician billings, visits to ambulatory care, hospital discharges, and the Activity Level Reporting database, which contains billings for all cancer-related care in Ontario [21]. Time to treatment initiation was the time between the date of cancer diagnosis and the receipt of any treatment for cancer, including surgery, chemotherapy, or radiation. This was represented with an ordinal variable, separated into twelve 3-month intervals of from the date of cancer diagnosis, with the twelfth interval including those who initiated treatment ≥ 3 years from cancer diagnosis and those who did not receive treatment. For sensitivity analyses, we derived three binary variables representing time to treatment initiation before or after 1, 3, and 6 months from cancer diagnosis.

### Outcome

The outcome of interest was the person-time from the date of cancer diagnosis to death from any cause. Mortality data were obtained from the Registered Persons Database (RPDB), which contains sociodemographic data for all Ontario residents [22].

### Data analysis

Descriptive characteristics of the analytic sample were summarized using frequencies, proportions, and standardized differences. We produced Kaplan-Meier curves following cancer diagnosis, stratified by stage at diagnosis, and compared people with NAPD and those without NAPD to illustrate differences in time to death from any cause between these groups.

### Analytic framework

Due to post-exposure confounding of the effect of stage at diagnosis on time to treatment initiation, we decomposed the randomized interventional analogue of total causal effect (RIATCE) into randomized interventional analogues of natural direct effects (RIANDE) and indirect effects (RIANIE), using methods developed by VanderWeele et al. [12]. In this approach, the RIANIE is described as the ratio of hazards of mortality following cancer diagnosis between people with the exposure if they had a randomly selected value of the mediator from the distribution of those exposed and the hazards of the outcome in the exposed group if they had a value of the mediator randomly selected from the distribution of the unexposed.

### Analytic implementation

The decomposition was performed in three separate mediation analyses using Cox proportional hazards models of mortality following cancer diagnosis, whereby stabilized inverse probability weights (sIPW) were used to adjust for confounders of each relationship according to the DAG in Fig. 1. The first analysis estimated the RIATCE, while the second and third estimated the RIANIE and RIANDE with stage at diagnosis then time to treatment initiation as mediators, respectively. This third analysis also adjusted for post-exposure by stage at diagnosis, which was assumed to be affected by diagnosis with NAPD on time to treatment initiation and mortality following cancer diagnosis. A detailed description of model specifications and the calculation of stabilized weights can be found in the supplemental materials.

The proportion mediated by stage at diagnosis and time to treatment initiation were calculated by dividing their respective RIANIE by the RIATCE, in terms of log-odds-ratios, and presented as a percentage.

The analyses were also stratified by primary tumour location and sex. Due to small cell counts for stage at diagnosis and time to treatment initiation when stratified by primary tumour location, we were restricted to performing these stratified analyses using binary definitions for mediators, as in our sensitivity analysis.

### Sensitivity analyses

We conducted sensitivity analyses to assess the robustness of our findings. The same analyses were run with alternative mediator definitions including binary variables for stage at diagnosis and time to treatment initiation to assess the effect of different operationalization of the mediators on effect estimates. For the sensitivity analysis with binary variables, stage at diagnosis was represented as either local/regional (I/II) or advanced (III/IV) and a binary variable was used to indicate whether someone received treatment within or following 6 months from cancer diagnosis. To explore the impact of excluding missing stage at diagnosis on the results, we performed the same analysis with binary variables for our mediators, but including those with unknown or missing stage at diagnosis in the advanced stage group (III/IV/missing). Confirmed by the Kaplan-Meier curve in the supplemental material, prior research suggests that people with missing stage at diagnosis have mortality risk intermediate to that of people diagnosed with early and advanced stage cancer [6, 23]. As such, for this sensitivity analysis, we included a level for unknown or missing stage which was intermediate to stages II and III, ordered as follows: I, II, unknown/missing, III, IV. To determine the impact of cutoff point selection for the binary variable of time to treatment initiation, we performed analyses with time to treatment initiation dichotomized at three months rather than six. Other sensitivity analyses explored alternative structures to the DAG in Fig. 1, such as those presented in Supplementary Fig. 1. All analyses were performed using SAS 9.4 [24], and results are presented as hazard ratios (HR) with corresponding 95% confidence intervals (CI).

## Results

The characteristics of the analytic sample are presented in Table 1. Between 1-January-2007 and 31-December-2019, there were 2,196 people with NAPD and 9,726 people without NAPD who were diagnosed with cancer. People with NAPD had a 66% greater adjusted hazard of death than people without NAPD following cancer diagnosis (HR = 1.66, 95%CI = 1.55,1.78).

### Mediation analysis

**Fig. 2:**
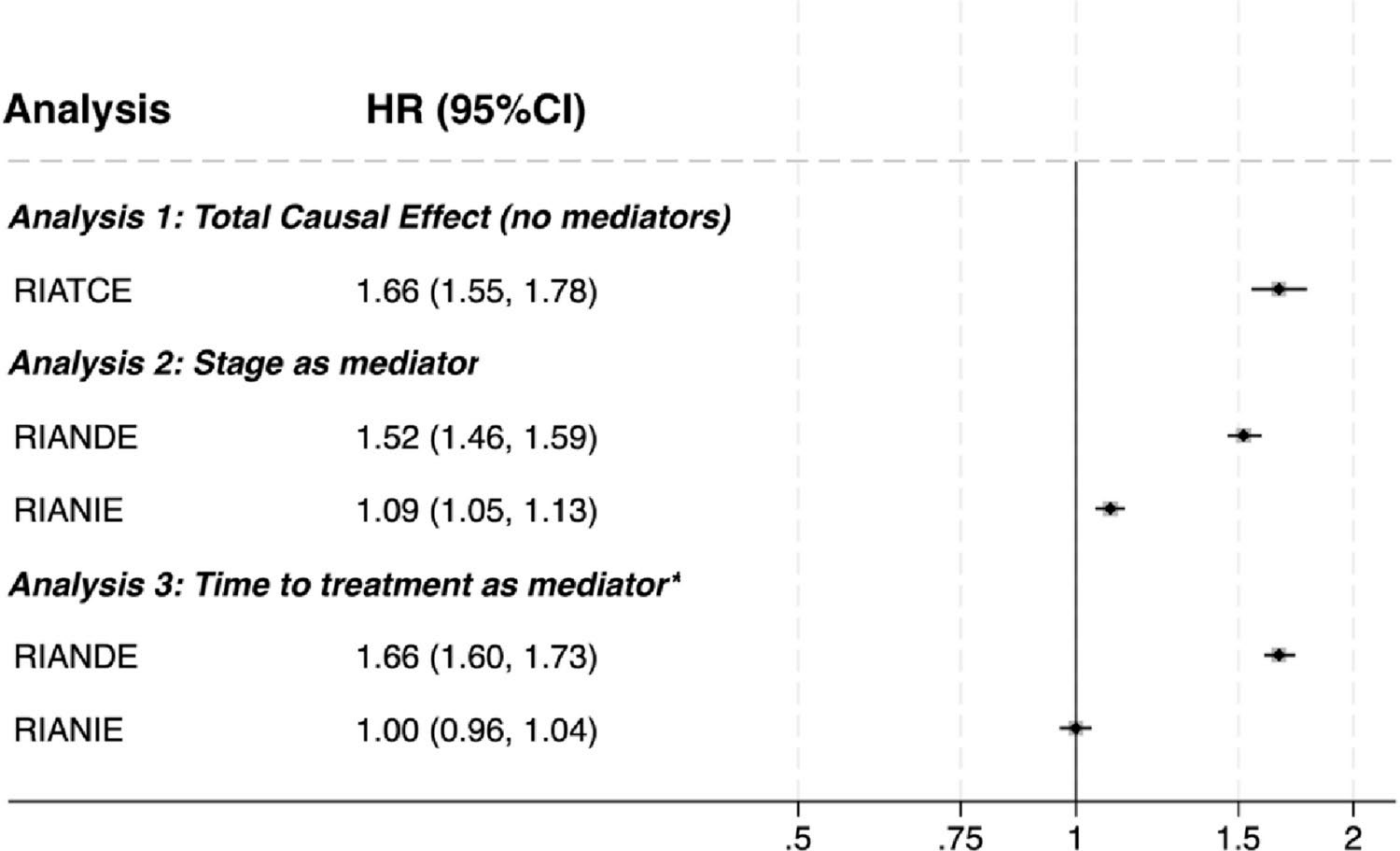
Results of the effect decomposition using stabilized inverse probability-weighted Cox proportional hazards models of diagnosis with NAPD on hazards of mortality following cancer diagnosis ^*^adjusting for stage at diagnosis as a postexposureconfounder

The results of the effect decomposition are displayed in Fig. 2. The estimate of the RIANIE for the effect of NAPD on the hazard of death following cancer diagnosis mediated through stage at diagnosis was 1.09 (95%CI = 1.05,1.13). The estimated proportion of the RIATCE, mediated by stage at diagnosis was 16.2%. The estimate of the RIANIE for the effect of NAPD on mortality following cancer diagnosis, mediated through time to treatment initiation was 1.00 (95%CI = 0.96,1.04). The estimated proportion of the RIATCE, mediated by time to treatment initiation is 0%.

**Fig. 3:**
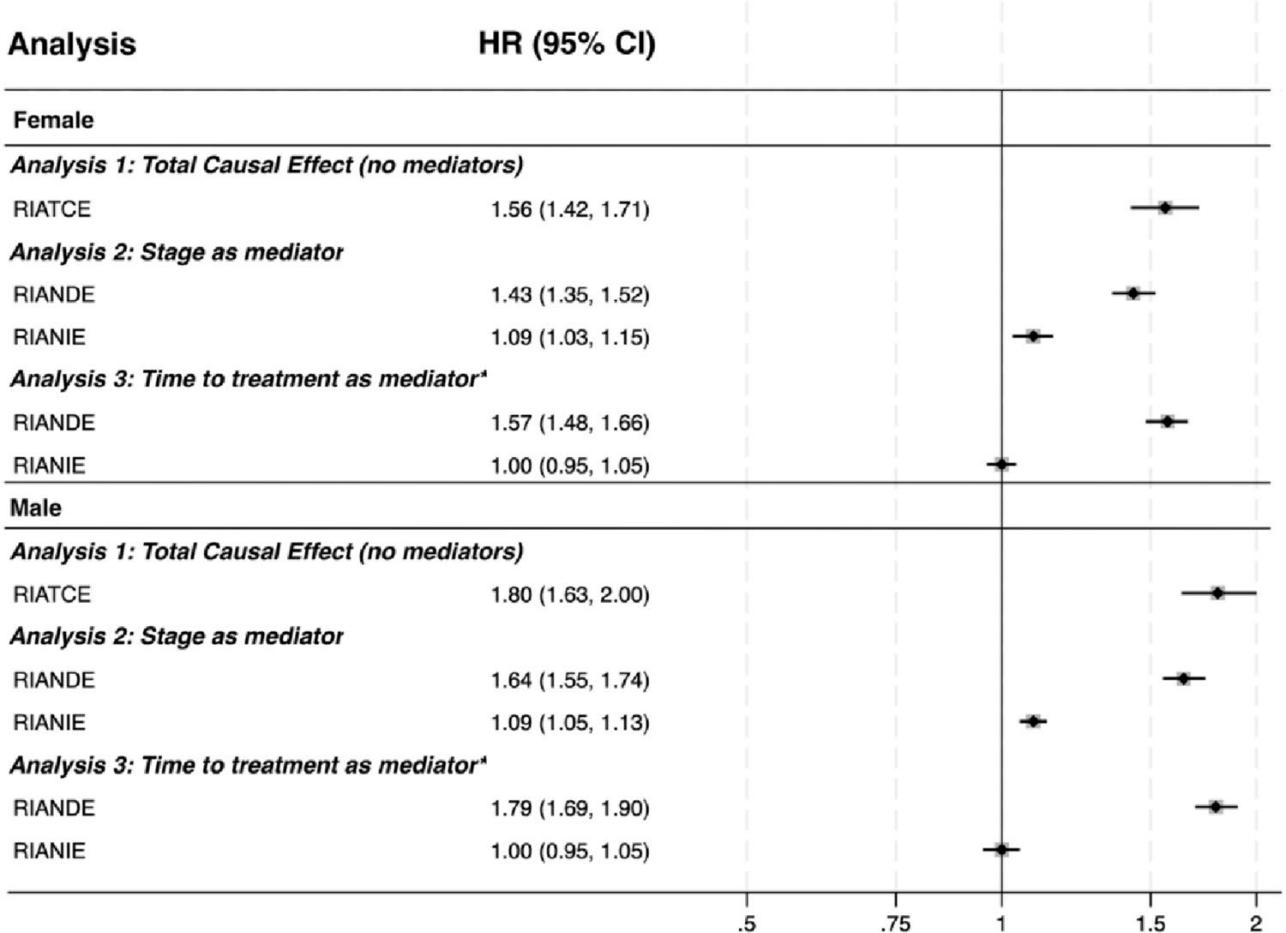
Results of the effect decomposition using stabilized inverse probability-weighted Cox proportional hazards models of diagnosis with NAPD on hazards of mortality following cancer diagnosis stratified by sex ^*^adjusting for stage at diagnosis as a postexposureconfounder

The results of the analysis stratified by sex can be found in Fig. 3. Males with NAPD had an 80% greater adjusted hazard of death than males without NAPD following cancer diagnosis (HR = 1.80, 95%CI = 1.63,2.00). Among males, the estimate of the RIANIE for the effect of NAPD on the hazard of death following cancer diagnosis mediated through stage at diagnosis was 1.09 (95%CI = 1.04,1.15). The estimate of the RIANIE for the effect of NAPD on mortality following cancer diagnosis, mediated through time to treatment initiation among males was 1.00 (95%CI = 0.95,1.05).

Females with NAPD had a 58% greater adjusted hazard of death than females without NAPD following cancer diagnosis (HR = 1.56, 95%CI = 1.42,1.71). Among females, the estimate of the RIANIE for the effect of NAPD on the hazard of death following cancer diagnosis mediated through stage at diagnosis was 1.09 (95%CI = 1.03,1.15). The estimate of the RIANIE for the effect of NAPD on mortality following cancer diagnosis, mediated through time to treatment initiation among females was 1.00 (95%CI = 0.95,1.05).

The results of the analysis stratified by tumour location can be found in Supplemental Table 4. The estimates of RIANIE through stage at diagnosis and time to treatment initiation were similar across most sites. The results of the sensitivity analyses can be found in Supplementary Table 5. Using our binary variables for stage at diagnosis and treatment initiation within 1, 3, 6 months of cancer diagnosis did not change results. Including unknown or missing stage at diagnosis between stages II and III in the ordinal variable did not materially change results. Additionally, using a binary variable for stage at diagnosis and grouping unknown or missing stage with advanced stage cancer did not materially change results. Lastly, adjustment for ADG at the time of cancer diagnosis, in accordance with the DAG in Supplementary Fig. 1A, slightly attenuated the estimate of the RIATCE to 1.65 (95%CI = 1.55, 1.77), but did not materially change estimates of other effects.

## Discussion

To our knowledge, this is the first study to apply alternative approaches to estimate how stage at diagnosis and time to treatment initiation may mediate excess risk of mortality following cancer diagnosis for people with NAPD, relative to those without. People with NAPD who were randomly assigned to have a value of stage at diagnosis from the distribution of people with NAPD had a 9% greater risk of mortality compared to people with NAPD, had they been randomly assigned a value of stage at diagnosis from the distribution of people without NAPD. Additionally, people with NAPD who were randomly assigned a time to treatment initiation of someone with NAPD had the same mortality risk following cancer diagnosis as people with NAPD, had they been randomly assigned a time to treatment initiation from the distribution of people without NAPD.

Our results suggest that for all cancer sites, only a modest proportion of excess mortality following cancer diagnosis among people with NAPD, relative to people without NAPD, was mediated by stage at diagnosis, and none through time to treatment initiation. These findings were robust to modifications to mediator definitions and operationalization, and other sensitivity analyses. Our analyses are strengthened by a large sample with a long follow-up period using health administrative data in a universal healthcare system.

We hypothesized that stage at diagnosis and/or time to treatment initiation would partly mediate the effect of NAPD on mortality following cancer diagnosis; however, our results suggest that only a small portion of that effect is mediated by stage at diagnosis, while none of it is mediated by time to treatment initiation. Irwin et al. have proposed a model wherein patient, provider, and system factors influence disparities along the cancer care continuum, ultimately contributing to worse mortality and other outcomes [25]. Although stage at diagnosis and time to treatment initiation are important predictors of mortality following cancer diagnosis, they only represent two of many such factors.

As mentioned previously, comorbid illnesses among people with NAPD is another proposed mediator, as these are associated with greater mortality following cancer diagnosis [26–29]. Furthermore, people with NAPD are more likely to engage in behaviours such as smoking, sedentariness, and poor diet, which also contribute to greater physical health burden and worse health outcomes [25]. In addition, people with NAPD have a much higher risk of death from suicide and moderately increased risk of death from accidents [30]. Previously, we found that in addition to a greater risk of cancer-related mortality, people with NAPD had a greater risk of non-cancer related mortality following cancer diagnosis, relative to people without NAPD [3]. As such, we expected that differences in comorbidity would account for a substantial proportion of the gap in all-cause mortality. Presently, we were unable to validly estimate the indirect effects through physical illness burden as a mediator, given that we only measured physical illness burden at the time of cancer diagnosis. Thus, it is difficult to discern between indirect effects of NAPD mediated by physical illness and the confounding effects of physical illness resulting from factors which preceded, and were independent of, diagnosis with NAPD. However, adjusting for comorbid physical illness did not change our findings.

Time to treatment initiation is only one dimension of cancer care among both physician adherence to guidelines and patient adherence to treatment, both of which influence treatment outcomes [31, 32]. Presently, the contribution of these factors to the mortality gap following cancer diagnosis among people with NAPD remains unexplored. Previously, Mahar et al. found that people with severe psychiatric illnesses were less likely to receive guideline appropriate care for colorectal cancer, when compared with people with no history of mental illness [8]. Importantly, patient adherence to treatment is dependent on mental health treatment and symptom management. Irwin et al. identified lack of mental health treatment as the biggest predictor for disruptions in breast cancer treatment among people with schizophrenia, rather than lack of public insurance, cancer stage, or access to a family physician [33]. This highlights the importance of transdisciplinary care [34], which can improve a patient’s ability to provide consent, promote patient adherence, and improve outcomes of oncologic care [25]. Additionally, implementation of treatment managers, which coordinate treatment across domains, may improve cohesiveness of care.

Furthermore, although mortality is a prominent outcome in cancer care literature, health-related quality of life, treatment satisfaction, and cancer recurrence are other crucial considerations [33]. In many cases, physicians and patients may be forced to balance these outcomes, meaning that exploration of any single outcome may not reflect the entire picture. We are not aware of any studies examining these other outcomes following cancer diagnosis among people with NAPD.

### Limitations

The estimates presented in this study assume correct model specification of relations between the exposure, mediators, confounders, and outcome. Therefore, misspecifications and unmeasured confounding may bias estimates presented in this study.

The case definition for NAPD has a high sensitivity and low specificity [14], which may have misclassified some people as having NAPD; however, this is expected to be non-differential. We did not exclude people with affective psychoses or other serious mental illnesses from the comparison group; therefore, effects may be underestimated.

Our study is limited by the billing data available in administrative databases held by ICES. Neighbourhood-level income may serve as a poor proxy for individual-level income [35]. We were also unable to adjust for confounding by occupation, level of education, ethnicity, genetics, lifestyle, and many other factors. Furthermore, neighbourhood-level income was only measured during census years, potentially misclassifying income across census intervals and resulting in residual confounding.

## Conclusions

Our findings suggest that stage at diagnosis plays a relatively small role in mediating excess risk of mortality following cancer diagnosis among people with NAPD, whereas time to treatment initiation does not mediate the effect. The excess mortality risk is likely mediated by other patient, provider, and system-related factors in cancer care, including health and behavioural factors. Future research should examine these factors to identify other points of intervention to target via treatment guidelines and integrated transdisciplinary care. Furthermore, we encourage future studies to explore disparities in treatment compliance and health related quality of life following diagnosis with cancer for people with psychosis.

**Table 1:** Descriptive characteristics of cohort members, by diagnosis with non-affective psychotic disorder

Characteristic		People with NAPD (*N* = 2,196)	People without NAPD (*N* = 9,726)	Total (*N* = 11,922)
Age	Mean (SD)	58.9 (9.4)	59.4 (9.9)	
	15 – 19	<10	<6	<20
	20 – 29	14 (0.6)	47 (0.5)	61 (0.5)
	30 – 39	68 (3.1)	367 (3.8)	435 (3.7)
	40 – 49	267 (12.2)	1083 (11.1)	1350 (11.3)
	50 – 59	687 (31.3)	2892 (29.7)	3579 (30.0)
	60 – 69	908 (41.4)	3903 (40.1)	4811 (40.4)
	70 – 79	248 (11.3)	1411 (14.5)	1659 (13.9)
	80 – 85	<10	<30	<27
Sex	Female	1349 (61.4)	5220 (53.7)	6569 (55.1)
	Male	847 (38.6)	4506 (46.3)	5353 (44.9)
Neighbourhood-level income quintile	1 (lowest)	757 (34.5)	1698 (17.5)	2455 (20.6)
	2	464 (21.1)	1960 (20.2)	2424 (20.3)
	3	379 (17.3)	1895 (19.5)	2274 (19.1)
	4	320 (14.6)	1997 (20.5)	2317 (19.4)
	5	276 (12.6)	2176 (22.4)	2452 (20.6)
Rurality of residence	Urban	1986 (90.4)	8353 (85.9)	10,339 (86.7)
	Rural	210 (9.6)	1373 (14.1)	1583 (13.3)
Access to family physician	No	134 (6.1)	413 (4.3)	547 (4.6)
	Yes	2062 (93.9)	9313 (95.8)	11,375 (95.4)
Psychiatric diagnosis	Schizophrenia spectrum disorder	862 (39.3)	--	862 (39.3)
	Psychosis NOS	1334 (60.8)	--	1334 (60.8)
Stage at cancer diagnosis	I	697 (31.7)	3273 (33.7)	3970 (33.3)
	II	533 (24.3)	2725 (28.0)	3258 (27.3)
	III	423 (19.3)	1809 (18.6)	2232 (18.7)
	IV	543 (24.7)	1919 (19.7)	2462 (20.7)
Received treatment within 6 months	Yes	1345 (61.3)	5709 (58.7)	7054 (59.2)
	No	851 (38.8)	4017 (41.3)	4868 (40.8)
Cancer Site	Bladder	21 (1.0)	70 (0.7)	91 (0.8)
	Brain	<6	<10	7 (0.1)
	Breast	578 (26.3)	2302 (23.7)	2880 (24.2)
	Cervix	26 (1.2)	113 (1.2)	139 (1.2)
	Colorectal	297 (13.5)	1279 (13.2)	1576 (13.2)
	Esophagus	24 (1.1)	77 (0.8)	101 (0.9)
	Hodgkin’s	11 (0.5)	33 (0.3)	44 (0.4)
	Kidney	32 (1.5)	146 (1.5)	178 (1.5)
	Larynx	10 (0.5)	74 (0.8)	84 (0.7)
	Leukemia	<6	<20	21 (0.2)
	Liver	16 (0.7)	77 (0.8)	93 (0.8)
	Lung	455 (20.7)	1357 (14.0)	1812 (15.2)
	Melanoma	49 (2.2)	320 (3.3)	369 (3.1)
	Non-Hodgkin	56 (2.6)	239 (2.5)	295 (2.5)
	Oral cavity	75 (3.4)	308 (3.2)	383 (3.2)
	Ovary	34 (1.6)	199 (2.1)	233 (2.0)
	Pancreas	29 (1.3)	172 (1.8)	201 (1.7)
	Prostate	205 (9.3)	1759 (18.1)	1964 (16.5)
	Stomach	18 (0.8)	115 (1.2)	133 (1.1)
	Testis	24 (1.1)	98 (1.0)	122 (1.0)
	Thyroid	41 (1.9)	248 (2.6)	289 (2.4)
	Uterus	115 (5.2)	407 (4.2)	522 (4.4)
	Other	76 (3.5)	309 (3.2)	385 (3.2)

## Supplementary Information

Below is the link to the electronic supplementary material.

Supplementary Material 1

## Data Availability

The dataset from this study is held securely in coded form at ICES. While legal data sharing agreements between ICES and data providers (e.g., healthcare organizations and government) prohibit ICES from making the dataset publicly available, access may be granted to those who meet pre-specified criteria for confidential access, available at www.ices.on.ca/DAS (email: das@ices.on.ca). The full dataset creation plan and underlying analytic code are available from the authors upon request, understanding that the computer programs may rely upon coding templates or macros that are unique to ICES and are therefore either inaccessible or may require modification.

## Declarations

MS received honoraria/has been a consultant for Angelini, AbbVie, Boehringer Ingelheim, Lundbeck, and Otsuka. The other authors have no disclosures to make.
