# Supplementary material for: Mortality following cancer diagnosis among people with non-affective psychoses: mediation by stage at diagnosis and time to treatment initiation
Source: Soc Psychiatry Psychiatr Epidemiol. 2026 Mar 5;61(7):1227–36. doi: 10.1007/s00127-026-03063-x (PMC13424192; doi:10.1007/s00127-026-03063-x)
Supplement: Supplementary file 1 — Supplementary Material 1 [file 127_2026_3063_MOESM1_ESM.docx]

SUPPLEMENTAL MATERIAL

Supplemental Table 1: The RECORD statement – checklist of items, extended from the STROBE statement, that should be reported in observational studies using routinely collected health data

|  | **Item No.** | **STROBE items** | **Location in manuscript where items are reported** | **RECORD items** | **Location in manuscript where items are reported** |
| --- | --- | --- | --- | --- | --- |
| **Title and abstract** | | | | | |
|  | 1 | (a) Indicate the study’s design with a commonly used term in the title or the abstract (b) Provide in the abstract an informative and balanced summary of what was done and what was found | Page 2 | RECORD 1.1: The type of data used should be specified in the title or abstract. When possible, the name of the databases used should be included.  RECORD 1.2: If applicable, the geographic region and timeframe within which the study took place should be reported in the title or abstract.  RECORD 1.3: If linkage between databases was conducted for the study, this should be clearly stated in the title or abstract. | Page 1  Page 2  Page 2 |
| **Introduction** | | | | | |
| Background rationale | 2 | Explain the scientific background and rationale for the investigation being reported | Page 3 |  |  |
| Objectives | 3 | State specific objectives, including any prespecified hypotheses | Page 3 |  |  |
| **Methods** | | | | | |
| Study Design | 4 | Present key elements of study design early in the paper | Page 4 |  |  |
| Setting | 5 | Describe the setting, locations, and relevant dates, including periods of recruitment, exposure, follow-up, and data collection | Page 4 |  |  |
| Participants | 6 | *(a) Cohort study* - Give the eligibility criteria, and the sources and methods of selection of participants. Describe methods of follow-up  *Case-control study* - Give the eligibility criteria, and the sources and methods of case ascertainment and control selection. Give the rationale for the choice of cases and controls  *Cross-sectional study* - Give the eligibility criteria, and the sources and methods of selection of participants  *(b) Cohort study* - For matched studies, give matching criteria and number of exposed and unexposed  *Case-control study* - For matched studies, give matching criteria and the number of controls per case | Pages 4  Page 4 | RECORD 6.1: The methods of study population selection (such as codes or algorithms used to identify subjects) should be listed in detail. If this is not possible, an explanation should be provided.  RECORD 6.2: Any validation studies of the codes or algorithms used to select the population should be referenced. If validation was conducted for this study and not published elsewhere, detailed methods and results should be provided.  RECORD 6.3: If the study involved linkage of databases, consider use of a flow diagram or other graphical display to demonstrate the data linkage process, including the number of individuals with linked data at each stage. | Page 4  Page 4  Appendix A4 |
| Variables | 7 | Clearly define all outcomes, exposures, predictors, potential confounders, and effect modifiers. Give diagnostic criteria, if applicable. | Page 5 | RECORD 7.1: A complete list of codes and algorithms used to classify exposures, outcomes, confounders, and effect modifiers should be provided. If these cannot be reported, an explanation should be provided. | Appendix A3 |
| Data sources/ measurement | 8 | For each variable of interest, give sources of data and details of methods of assessment (measurement).  Describe comparability of assessment methods if there is more than one group | Appendix A3  Page 5 |  |  |
| Bias | 9 | Describe any efforts to address potential sources of bias | Page 4 |  |  |
| Study size | 10 | Explain how the study size was arrived at | Appendix A4 |  |  |
| Quantitative variables | 11 | Explain how quantitative variables were handled in the analyses. If applicable, describe which groupings were chosen, and why | Page 5 |  |  |
| Statistical methods | 12 | (a) Describe all statistical methods, including those used to control for confounding  (b) Describe any methods used to examine subgroups and interactions  (c) Explain how missing data were addressed  (d) *Cohort study* - If applicable, explain how loss to follow-up was addressed  *Case-control study* - If applicable, explain how matching of cases and controls was addressed  *Cross-sectional study* - If applicable, describe analytical methods taking account of sampling strategy  (e) Describe any sensitivity analyses | Pages 6 –7  Pages 6 –7  Pages 6 –7  Pages 6 –7  Pages 6 –7 |  |  |
| Data access and cleaning methods |  | .. |  | RECORD 12.1: Authors should describe the extent to which the investigators had access to the database population used to create the study population.  RECORD 12.2: Authors should provide information on the data cleaning methods used in the study. | Pages 6 –7  Pages 6 –7 |
| Linkage |  | .. |  | RECORD 12.3: State whether the study included person-level, institutional-level, or other data linkage across two or more databases. The methods of linkage and methods of linkage quality evaluation should be provided. | Pages 7 – 8 |
| **Results** | | | | | |
| Participants | 13 | (a) Report the numbers of individuals at each stage of the study (*e.g.*, numbers potentially eligible, examined for eligibility, confirmed eligible, included in the study, completing follow-up, and analysed)  (b) Give reasons for non-participation at each stage.  (c) Consider use of a flow diagram | Page 7 and appendix A4  Page 7 and appendix A4 | RECORD 13.1: Describe in detail the selection of the persons included in the study (*i.e.,* study population selection) including filtering based on data quality, data availability and linkage. The selection of included persons can be described in the text and/or by means of the study flow diagram. | Page 7 and appendix A4 |
| Descriptive data | 14 | (a) Give characteristics of study participants (*e.g.*, demographic, clinical, social) and information on exposures and potential confounders  (b) Indicate the number of participants with missing data for each variable of interest  (c) *Cohort study* - summarise follow-up time (*e.g.*, average and total amount) | Pages 7 – 8 and table 2  Appendix A4  Page 7 &Figure 1 |  |  |
| Outcome data | 15 | *Cohort study* - Report numbers of outcome events or summary measures over time  *Case-control study* - Report numbers in each exposure category, or summary measures of exposure  *Cross-sectional study* - Report numbers of outcome events or summary measures | Page 7 |  |  |
| Main results | 16 | (a) Give unadjusted estimates and, if applicable, confounder-adjusted estimates and their precision (e.g., 95% confidence interval). Make clear which confounders were adjusted for and why they were included  (b) Report category boundaries when continuous variables were categorized  (c) If relevant, consider translating estimates of relative risk into absolute risk for a meaningful time period | Figure 1 |  |  |
| Other analyses | 17 | Report other analyses done—e.g., analyses of subgroups and interactions, and sensitivity analyses | Pages 8 – 9  Appendix A5 |  |  |
| **Discussion** | | | | | |
| Key results | 18 | Summarise key results with reference to study objectives | Pages 9 – 12 |  |  |
| Limitations | 19 | Discuss limitations of the study, considering sources of potential bias or imprecision. Discuss both direction and magnitude of any potential bias | Page 12 | RECORD 19.1: Discuss the implications of using data that were not created or collected to answer the specific research question(s). Include discussion of misclassification bias, unmeasured confounding, missing data, and changing eligibility over time, as they pertain to the study being reported. | Pages 9 –13 |
| Interpretation | 20 | Give a cautious overall interpretation of results considering objectives, limitations, multiplicity of analyses, results from similar studies, and other relevant evidence | Page 13 |  |  |
| Generalisability | 21 | Discuss the generalisability (external validity) of the study results | Page 13 |  |  |
| **Other Information** | | | | | |
| Funding | 22 | Give the source of funding and the role of the funders for the present study and, if applicable, for the original study on which the present article is based | Pages 13 –14 |  |  |
| Accessibility of protocol, raw data, and programming code |  | .. |  | RECORD 22.1: Authors should provide information on how to access any supplemental information such as the study protocol, raw data, or programming code. | Page 14 |

Supplemental Table 2: Databases accessed at ICES

| **Database** | - **Description** |
| --- | --- |
| Registered Persons Database (RPDB) | - The Registered Persons Database (RPDB) contains socio-demographic data for all Ontario residents registered for the Ontario Health Insurance Plan (OHIP) (Ontario Ministry of Health and Long Term Care 2012). Information in the RPDB includes date of birth, residential information, and dates of last contact with the healthcare system (Ontario Ministry of Health and Long Term Care 2012). |
| Canadian Institute for Health Information Discharge Abstract Database (CIHI-DAD) | - The Canadian Institute for Health Information Discharge Abstract Database (CIHI-DAD) contains data regarding separations from acute care, including discharges, deaths, sign-outs, and transfers, which are collected directly from acute care facilities and public health departments (Canadian Institute for Health Information 2019). Each abstract contains data pertaining to hospital activity for each separation, including diagnostic, intervention, patient demographic, and administrative information (Canadian Institute for Health Information 2019). |
| National Ambulatory Care Reporting System (NACRS) | - The National Ambulatory Care Reporting System (NACRS) is a database created by CIHI for the purposes of capturing information regarding client visits to facility and community-based ambulatory care (Canadian Institute for Health Information 2011). NACRS contains demographic, clinical, administrative, financial, and service-specific information for emergency department visits, day surgery procedures, diagnostic imaging, and clinic visits (Canadian Institute for Health Information 2011). |
| OHIP Claims Database | - The OHIP Claims Database contains fee-for-service claims, as well as non-fee-associated (shadow billing) claims for physicians working under other payment models, submitted by physicians for health services provided to Ontario residents under OHIP (Ontario Ministry of Health and Long Term Care 2012). For each claim, information regarding date and location of service, type of service provider, diagnosis, and a service fee code is provided (Goel *et al.* 1996). OHIP covers approximately 97% of all Ontario residents (Black *et al.* 2018) |
| Ontario Cancer Registry (OCR) | - The Ontario Cancer Registry (OCR) compiles data on all cases of cancer diagnosed among Ontario residents (Prodhan *et al.* 2016). This registry allows accurate reporting of cancer incidence, prevalence, and mortality. This reporting is facilitated through the compilation of data from the CIHI DAD and National Ambulatory Care Reporting System (NACRS) databases, as well data from regional cancer centres, pathology reports, death certificates, and out-of-province cancer diagnoses. |
| Primary Care Population dataset (PCPOP) | - The Primary Care Population dataset (PCPOP) includes all Ontario residents who have had contact with health services within any 7- to 9-year period, starting in the year 2000 (Slater *et al.* 2019). The PCPOP contains data on demographics, primary care, attachment status, and a number of variables specific to contact with healthcare services (Slater *et al.* 2019). |
| ICES Physician Database (IPDB) | - The ICES Physician Database (IPDB) contains demographic, specialty, and other information about physicians practicing in the province of Ontario (Care and Experience 2015). |
| Activity Level Reporting (ALR) | - The Activity Level Reporting (ALR) database contains billings for all cancer-related care in Ontario. The sensitivities and specificities are 92.0% and 99.3% for radiotherapy and 77.7% and 99.2% for chemotherapy, respectively (Weerasinghe *et al.* 2018). |

| Database | Qualifying Event |
| --- | --- |
| Discharge Abstract Database (DAD) | ≥ 1 primary discharge diagnosis of schizophrenia, schizoaffective disorder, schizophreniform, or psychosis not otherwise specified (ICD-9 codes 295.x or 298.x; ICD-10 codes F20, F25, or F29) |
| **OR** | |
| Ontario Health Insurance Plan (OHIP) Database | ≥ 2 OHIP billings within 24 months with a diagnostic code (DXCODE) for schizophrenia, schizoaffective disorder, schizophreniform, or psychosis not otherwise specified (ICD-9 codes 295.x or 298.x; ICD-10 codes F20, F25, or F29) |

Supplemental Table 3: Validated algorithm for identifying cases of non-affective psychotic disorders in ICES (Kurdyak *et al.* 2015)

ICD=International Classification of Diseases

**B**

**A**

**C**

Supplemental Figure : Directed acyclic graphs (DAGs) depicting proposed alternate causal structures

**Details of mediation analysis using randomized interventional analogues**

##### **Brief overview of effect decomposition using randomized interventional analogues of effects** ^80^

An alternative to the unidentifiable natural indirect effects in scenarios with post-exposure confounding of the M-O relationship are the randomized analogues of interventional effects (RIA), initially described by Didelez et al.^106^ and Geneletti,^107^ then adapted by Vanderweele et al.^80^ for implementation in mediation analyses with post-exposure confounding. An adaptation of this approach has also been demonstrated by Li et al.^82^ in the identification in the mediated effects of deprivation on BC mortality, mediated by stage at diagnosis and time to treatment initiation. To identify analogues of ‘interventional’ effects, mediators are set to a value which is randomly selected from the *distribution* of that mediator, conditioned on either exposure or non-exposure.^80^

Under this framework, the randomized interventional analogue of the total causal effect (RIATCE) of exposure *A* = *a*, relative to non-exposure *A* = *a**, on the outcome *Y,* is defined as:

$$\left( 1 \right) RIATCE= E(Y_{aG_{a}|C}) - E(Y_{a^{*}G_{a^{*}}|C})$$

Whereby, *G_a|C_* denotes a random selection of a value of the mediator from the distribution of the mediator among those with exposure level *a*, conditioning on covariates *C*; and therefore, *Y_aGa|C_* denotes the outcome that would be observed under given exposure *a* and *G_a|C_.* Similarly, *Y_aGa|C_* denotes the outcome that would be observed under given exposure *a** and a random selection of a value of the mediator among those with exposure *a**. Under this framework, the RIATCE can be decomposed into the randomized interventional analogues of natural direct and indirect effects (RIANDE and RIANIE). The RIANIE is defined by equation 2.^80^

The RIANIE contrasts the expected outcome of someone who is exposed (*a*) being randomly assigned a value from the distribution of *M* if they are exposed (*G_a|C_*), and the expected outcome of someone who is exposed (*a*) being randomly assigned a value of *M* from the distribution that *M* would take, given non-exposure (a*).The RIANDE contrasts the expected outcome of someone who is exposed (a) being randomly assigned a value from the distribution of M if they were unexposed (*G_a*|C_*), to the expected value of the outcome of someone who is unexposed (a*) being randomly assigned a value from the distribution of M given non-exposure (*G_a*|C_*), defined by equation 3.

As such, the decomposition of the RIATCE into the RIANIE and RIANDE is expressed by equation 4.

}

Vanderweele et al. stipulate the following assumptions required for the identification of the RIANIE,^80^ RIANDE, and RIATCE in scenarios involving multiple mediators and post-exposure confounding:

1. No unmeasured exposure-outcome confounding, conditional on covariates C, denoted by:
2. No unmeasured mediator outcome confounding, conditional on A, C, and L, denoted by:
3. No unmeasured exposure-mediator confounding, conditional on C, denoted by:

Under these assumptions, the RIANIE and RIANDE can be identified using the following 2 equations:^80^

Whereby, *l* denotes the post-exposure confounder of the mediator-outcome relationship. In the proposed study, the post-exposure confounder when considering the natural indirect effect through time to treatment initiation, is stage at diagnosis.

To contrast the effects of ‘counterfactual’ values of the exposure, mediator, and post-exposure confounder, this approach uses duplications of the observed dataset.^80^ Each duplication corresponds to each ‘counterfactual’ or hypothetical combination of values of exposure (*a*), mediator (*m*), and post-exposure confounder (*l*). The RIANIE and RIANDE can then be identified using the outcome models using the duplicated dataset, with weights calculated using the observed dataset.^80^ Regression equations are then used to predict the following terms: *P*(*a | c*), *P*(*l | a*, c*), and *P*(*m | l, a*, c*).^80^ These terms are then used in the calculation of weights for each observation, according to the following:

To decompose the RIATCE of diagnosis with NAPD on the hazards of mortality following cancer diagnosis, mediated through stage at diagnosis and time to treatment initiation, as illustrated by figure 1, we conducted 3 analyses: (1) the RIATCE of diagnosis with NAPD (*A=*1), relative to not being diagnosed with NAPD (A=0), on the hazards of mortality following cancer diagnosis (2) the RIANIE of diagnosis with NAPD (A=1) on the hazards of mortality following cancer diagnosis, relative to those without NAPD (A=0), mediated by stage at diagnosis; (3) the effect of diagnosis with NAPD (A=1), relative to not being diagnosed with NAPD (A=0), on the hazards of mortality following cancer diagnosis, mediated by time to treatment initiation. Each analysis requires the separate calculations of weights and duplications of the observed dataset.

#### Analysis 1: RIATCE of diagnosis with NAPD on mortality following cancer diagnosis

To compute weights to estimate the RIATCE, a logistic regression model of the odds of having a diagnosis of NAPD was set on our covariates measured at the time of cancer diagnosis. The resulting model was used to predict the propensity of each person having a diagnosis with NAPD, conditional on age at cancer diagnosis, sex assigned at birth, as well as income quintile, rurality of residence, and access to a family physician, at the time of cancer diagnosis. This propensity score was then used in the calculation of the stabilized weights by exposure (diagnosis with NAPD or not diagnosed with NAPD), according to the following weights in equations 7 and 8, respectively:

A Cox proportional model of the hazards of mortality following cancer diagnosis was then fit on diagnosis with NAPD, using the above weights.^80^ The resulting HR and 95% confidence interval associated with NAPD was then estimated the RIATCE of diagnosis with NAPD on the hazards of mortality following cancer diagnosis, adjusting for age at cancer diagnosis, sex assigned at birth, as well as income quintile, rurality of residence, and access to a family physician, at the time of cancer diagnosis. The RIATCE was presented as HR with 95% confidence intervals.

#### Analysis 2: effect of diagnosis with NAPD on mortality following cancer diagnosis, mediated by stage at diagnosis

To compute the weights in this analysis, we used 2 regression models: (1) *P*(*a | c*), logistic regression of having NAPD on age at cancer diagnosis, sex assigned at birth, as well as income quintile, rurality of residence, and access to a family physician, at the time of cancer diagnosis. and *P*(*m | a, c*), an ordinal logistic regression of stage at diagnosis on age at cancer diagnosis, sex assigned at birth, as well as income quintile, rurality of residence, and access to a family physician, at the time of cancer diagnosis and diagnosis with NAPD. The observed dataset was then duplicated with each duplication setting a new variable, a*, representing the counterfactual exposures, 0 or 1. Stabilized weights were calculated using the predicted odds of *a and m,* according to the following equation:

The 3 datasets (1 observed, 2 duplicates), along with their computed weights were then merged. To identify the RIANDE, a Cox proportional hazards model of mortality following cancer diagnosis was then fit on diagnosis with NAPD, using the stabilized weights in equation 9 for all observations with *a** = 0. To identify the RIANIE through stage at diagnosis, a Cox proportional hazards model of mortality following cancer diagnosis was fit on the counterfactual exposure a*, using the stabilized weights from equation 9 for all observations with NAPD (*a* = 1). The RIANIE and RIANDE were presented as HR and 95% confidence intervals.

#### Analysis 3: effect of diagnosis with NAPD on mortality following cancer diagnosis, mediated by time to treatment initiation

As mentioned previously, stage at diagnosis acts as a post-exposure confounder of the pathway between time to treatment initiation and mortality following cancer diagnosis; therefore, the weightings used in the models for analysis 3 incorporated propensity for the distribution of time to treatment initiation. For the calculation of weights in this analysis, we used 3 regression models: (1) *P*(*a | c*), logistic regression of having NAPD on age at cancer diagnosis, sex assigned at birth, as well as income quintile, rurality of residence, and access to a family physician, at the time of cancer diagnosis; (2) *P*(*l | a*, c*), an ordinal logistic regression of stage at diagnosis (I, II,III,IV) on age at cancer diagnosis, sex assigned at birth, as well as income quintile, rurality of residence, and access to a family physician, at the time of cancer diagnosis and diagnosis with NAPD; and (3) *P*(*m | l, a, c*), logistic regression of time to treatment initiation on age at cancer diagnosis, sex assigned at birth, as well as income quintile, rurality of residence, and access to a family physician, at the time of cancer diagnosis, diagnosis with NAPD, and stage at diagnosis. The observed dataset was then duplicated with each duplication setting a new variable, a*, representing the counterfactual exposure, to 0 or 1. Stabilized weights were calculated using the predicted odds of *a, m,* and *l,* according to the following equation:

The 3 datasets (1 observed, 2 duplicates), along with their computed weights were then merged. To identify the RIANDE, a Cox proportional hazards model of mortality following cancer diagnosis was then fit on diagnosis with NAPD, using the stabilized weights in equation 10 for all observations with *a** = 0. To identify the RIANIE through time to treatment initiation, a Cox proportional hazards model of mortality following cancer diagnosis was fit on the counterfactual exposure a*, using the stabilized weights from equation 10 for all observations with NAPD (*a* = 1). The RIANIE and RIANDE were presented as HR and 95% confidence intervals.


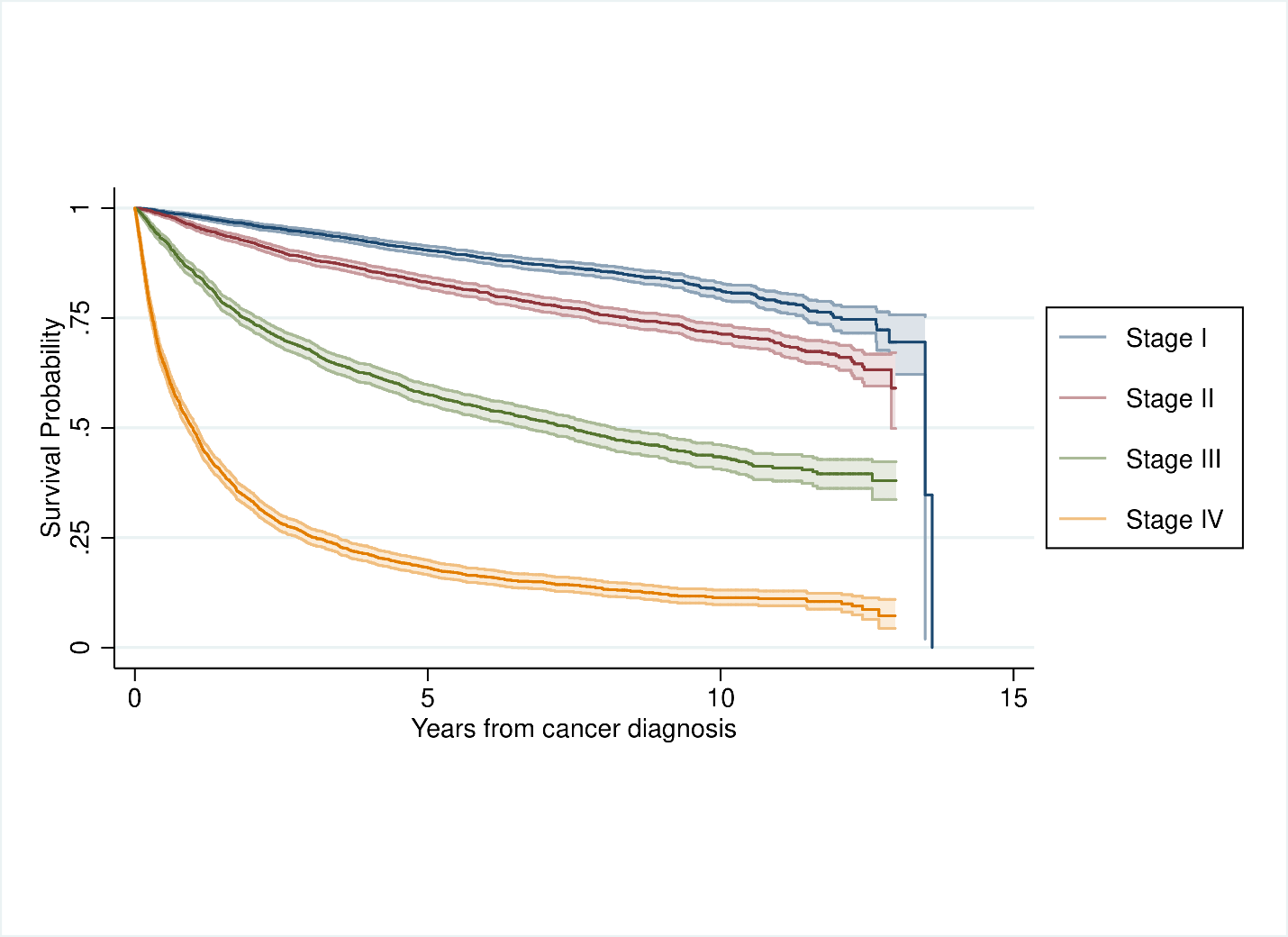


Supplemental Figure : Kaplan Meier survival curves following cancer diagnosis, stratified by stage at diagnosis

*
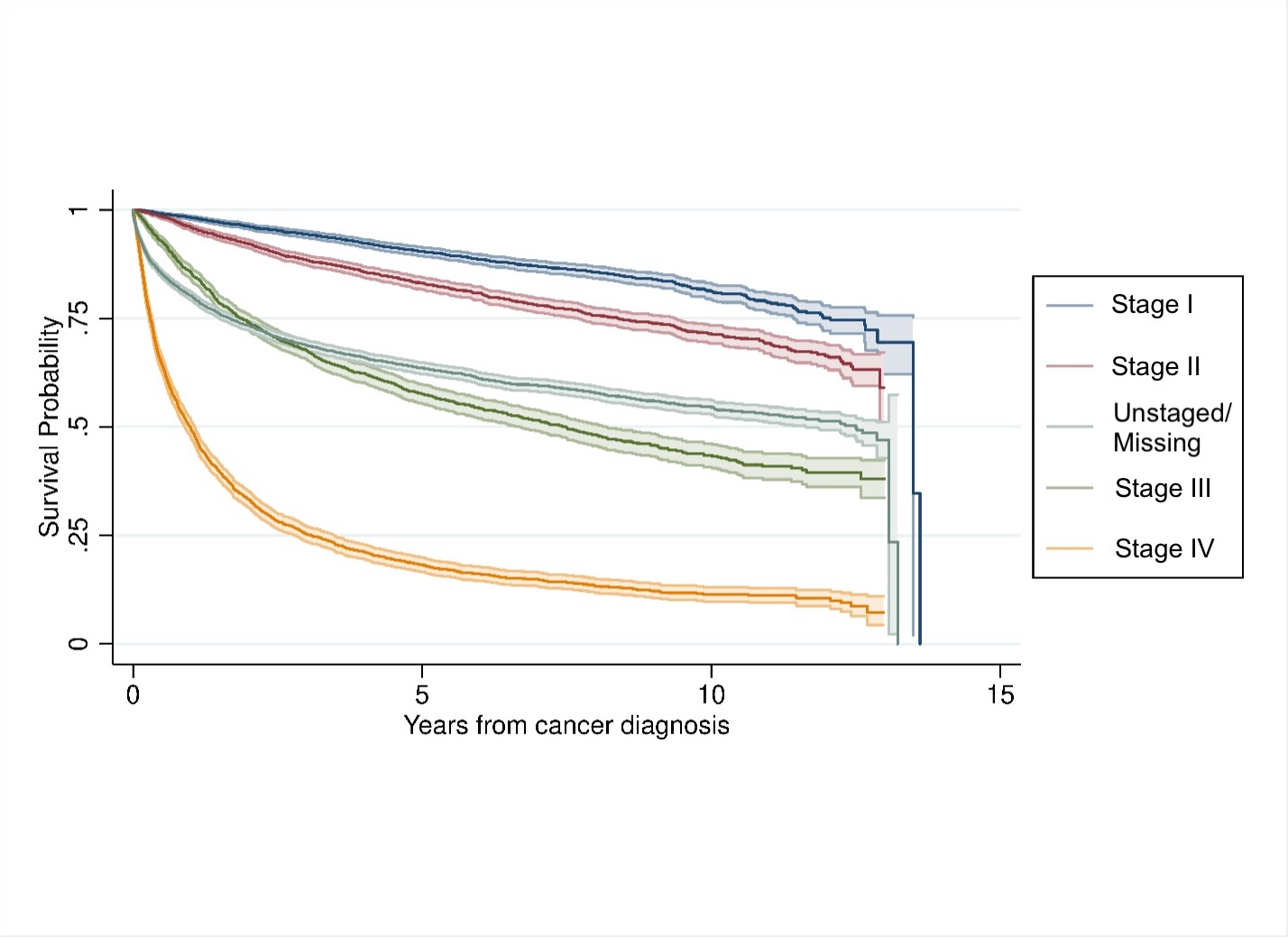
*

Supplemental Figure : Kaplan Meier survival curves following cancer diagnosis, stratified by stage at diagnosis and including unknown or missing stage at diagnosis

Supplemental Table : Results of the effect decomposition using stabilized inverse probability-weighted Cox proportional hazards models of mortality following cancer diagnosis on diagnosis with NAPD, stratified by primary tumour location.

| **Site** | **Mediator** | **Effect** | **HR (95% CI)** |
| --- | --- | --- | --- |
| Bladder | None | RIATCE | 1.08 (0.58, 2.02) |
|  | Stage | RIANIE | 0.97 (0.66, 1.43) |
|  |  | RIANDE | 0.82 (0.57, 1.20) |
|  | Treatment | RIANIE | 1.04 (0.70, 1.54) |
|  |  | RIANDE | 0.78 (0.53, 1.15) |
| Brain | None | RIATCE | 5.48 (0.34, 87.82) |
|  | Stage | RIANIE | 1.00 (0.06, 15.99) |
|  |  | RIANDE | 5.48 (0.34, 87.82) |
|  | Treatment | RIANIE | 1.00 (0.00, .) |
|  |  | RIANDE | 6.03 (0.00, .) |
| Breast | None | RIATCE | 2.33 (1.93, 2.80) |
|  | Stage | RIANIE | 1.09 (0.98, 1.20) |
|  |  | RIANDE | 2.09 (1.86, 2.36) |
|  | Treatment | RIANIE | 1.01 (0.92, 1.11) |
|  |  | RIANDE | 2.25 (2.00, 2.53) |
| Cervix | None | RIATCE | 1.00 (0.47, 2.15) |
|  | Stage | RIANIE | 1.00 (0.65, 1.54) |
|  |  | RIANDE | 0.91 (0.60, 1.40) |
|  | Treatment | RIANIE | 1.16 (0.74, 1.83) |
|  |  | RIANDE | 0.80 (0.51, 1.24) |
| Colorectal | None | RIATCE | 1.92 (1.60, 2.29)* |
|  | Stage | RIANIE | 1.08 (0.98, 1.19) |
|  |  | RIANDE | 1.72 (1.55, 1.92) |
|  | Treatment | RIANIE | 1.10 (0.99, 1.21) |
|  |  | RIANDE | 1.70 (1.52, 1.89) |
| Esophagus | None | RIATCE | 1.62 (0.97, 2.68) |
|  | Stage | RIANIE | 1.21 (0.87, 1.67) |
|  |  | RIANDE | 1.35 (0.98, 1.86) |
|  | Treatment | RIANIE | 1.07 (0.77, 1.49) |
|  |  | RIANDE | 1.48 (1.07, 2.04) |
| Hodgkins | None | RIATCE | 1.49 (0.29, 7.79) |
|  | Stage | RIANIE | 0.95 (0.28, 3.21) |
|  |  | RIANDE | 1.12 (0.35, 3.54) |
|  | Treatment | RIANIE | 0.68 (0.22, 2.13) |
|  |  | RIANDE | 1.66 (0.57, 4.89) |
| Kidney | None | RIATCE | 1.13 (0.63, 2.01) |
|  | Stage | RIANIE | 1.12 (0.82, 1.53) |
|  |  | RIANDE | 1.14 (0.83, 1.56) |
|  | Treatment | RIANIE | 1.07 (0.79, 1.46) |
|  |  | RIANDE | 1.19 (0.87, 1.62) |
| Larynx | None | RIATCE | 2.91 (1.25, 6.75)* |
|  | Stage | RIANIE | 0.95 (0.65, 1.40) |
|  |  | RIANDE | 3.23 (2.04, 5.11) |
|  | Treatment | RIANIE | 1.40 (0.94, 2.10) |
|  |  | RIANDE | 2.14 (1.33, 3.45) |
| Leukemia | None | RIATCE | 2.03 (0.43, 9.64) |
|  | Stage | RIANIE | 1.03 (0.13, 7.96) |
|  |  | RIANDE | 1.90 (0.36, 10.01) |
|  | Treatment | RIANIE | 0.88 (0.07, 11.53) |
|  |  | RIANDE | 3.87 (0.37, 40.92) |
| Liver | None | RIATCE | 0.56 (0.27, 1.13) |
|  | Stage | RIANIE | 0.55 (0.37, 0.84)* |
|  |  | RIANDE | 0.94 (0.65, 1.36) |
|  | Treatment | RIANIE | 0.95 (0.62, 1.44) |
|  |  | RIANDE | 0.54 (0.37, 0.78) |
| Lung | None | RIATCE | 1.23 (1.09, 1.39)* |
|  | Stage | RIANIE | 1.01 (0.94, 1.09) |
|  |  | RIANDE | 1.22 (1.13, 1.31) |
|  | Treatment | RIANIE | 1.02 (0.95, 1.10) |
|  |  | RIANDE | 1.20 (1.12, 1.30) |
| Melanoma | None | RIATCE | 2.10 (1.20, 3.69)* |
|  | Stage | RIANIE | 1.20 (0.88, 1.63) |
|  |  | RIANDE | 1.20 (0.85, 1.69) |
|  | Treatment | RIANIE | 1.21 (0.89, 1.66) |
|  |  | RIANDE | 1.18 (0.84, 1.66) |
| Non-Hodgkin | None | RIATCE | 1.61 (1.00, 2.58)* |
|  | Stage | RIANIE | 1.09 (0.85, 1.39) |
|  |  | RIANDE | 1.61 (1.21, 2.14) |
|  | Treatment | RIANIE | 1.01 (0.79, 1.28) |
|  |  | RIANDE | 1.74 (1.31, 2.31) |
| Oral | None | RIATCE | 1.73 (1.22, 2.45)* |
|  | Stage | RIANIE | 1.01 (0.84, 1.22) |
|  |  | RIANDE | 1.82 (1.47, 2.25) |
|  | Treatment | RIANIE | 0.91 (0.76, 1.09) |
|  |  | RIANDE | 2.03 (1.65, 2.51) |
| Ovary | None | RIATCE | 1.56 (0.95, 2.56) |
|  | Stage | RIANIE | 1.23 (0.95, 1.58) |
|  |  | RIANDE | 1.34 (1.03, 1.75) |
|  | Treatment | RIANIE | 0.97 (0.76, 1.24) |
|  |  | RIANDE | 1.75 (1.35, 2.28) |
| Pancreas | None | RIATCE | 1.79 (1.18, 2.71)* |
|  | Stage | RIANIE | 1.01 (0.82, 1.23) |
|  |  | RIANDE | 1.83 (1.46, 2.29) |
|  | Treatment | RIANIE | 1.02 (0.83, 1.25) |
|  |  | RIANDE | 1.71 (1.36, 2.15) |
| Prostate | None | RIATCE | 2.30 (1.71, 3.08)* |
|  | Stage | RIANIE | 1.01 (0.90, 1.14) |
|  |  | RIANDE | 2.33 (2.01, 2.71) |
|  | Treatment | RIANIE | 1.03 (0.91, 1.16) |
|  |  | RIANDE | 2.30 (1.98, 2.67) |
| Stomach | None | RIATCE | 1.31 (0.74, 2.30) |
|  | Stage | RIANIE | 0.84 (0.61, 1.16) |
|  |  | RIANDE | 1.10 (0.82, 1.48) |
|  | Treatment | RIANIE | 0.94 (0.68, 1.30) |
|  |  | RIANDE | 0.97 (0.72, 1.32) |
| Testis | None | RIATCE | 2.11 (0.39, 11.55) |
|  | Stage | RIANIE | 1.13 (0.55, 2.35) |
|  |  | RIANDE | 3.24 (1.11, 9.47) |
|  | Treatment | RIANIE | 0.89 (0.45, 1.76) |
|  |  | RIANDE | 4.16 (1.46, 11.81) |
| Thyroid | None | RIATCE | 0.83 (0.19, 3.61) |
|  | Stage | RIANIE | 1.12 (0.41, 3.06) |
|  |  | RIANDE | 0.37 (0.16, 0.88) |
|  | Treatment | RIANIE | 1.02 (0.38, 2.74) |
|  |  | RIANDE | 0.41 (0.18, 0.94) |
| Uterus | None | RIATCE | 1.36 (0.91, 2.02) |
|  | Stage | RIANIE | 1.18 (0.95, 1.47) |
|  |  | RIANDE | 1.36 (1.06, 1.74) |
|  | Treatment | RIANIE | 1.01 (0.82, 1.25) |
|  |  | RIANDE | 1.58 (1.24, 2.01) |
| Other | None | RIATCE | 0.99 (0.67, 1.47) |
|  | Stage | RIANIE | 0.87 (0.71, 1.07) |
|  |  | RIANDE | 1.18 (0.96, 1.46) |
|  | Treatment | RIANIE | 1.00 (0.81, 1.24) |
|  |  | RIANDE | 1.02 (0.83, 1.27) |

CI=confidence interval, HR=hazard ratio, NAPD=non-affective psychotic disorder

^†^Adjusting for age, sex, neighbourhood-level income quintile, rurality of residence, and access to a family physician

*Statistically significant (p<0.05)

Supplemental Table : Results of sensitivity analyses of effect decomposition using stabilized inverse probability-weighted Cox proportional hazards models of diagnosis with NAPD on hazards of mortality following cancer diagnosis

| **Sensitivity Analysis** | **Mediator** | **Effect** | **HR (95% CI)*** |
| --- | --- | --- | --- |
| Including unknown/missing stage at diagnosis as stage between stages II and III (I, II, missing, III, IV) | None | RIATCE | 1.68 (1.60, 1.77) |
|  | Stage | RIANIE | 1.06 (1.03, 1.09) |
|  |  | RIANDE | 1.58 (1.53, 1.63) |
|  | Treatment | RIANIE | 1.01 (0.98, 1.04) |
|  |  | RIANDE | 1.66 (1.61, 1.72) |
| Binary definitions of mediators grouping missing stage with advanced stage (early stage vs. advanced/missing) | None | RIATCE | 1.68 (1.60, 1.77) |
|  | Stage | RIANIE | 1.06 (1.03, 1.09) |
|  |  | RIANDE | 1.58 (1.53, 1.63) |
|  | Treatment | RIANIE | 1.01 (0.98, 1.04) |
|  |  | RIANDE | 1.66 (1.61, 1.72) |
| Adjusting for ADG as a confounder (DAG in Supplemental Figure 1A) | None | RIATCE | 1.62 (1.51, 1.74) |
|  | Stage | RIANIE | 1.10 (1.05, 1.14) |
|  |  | RIANDE | 1.48 (1.42, 1.54) |
|  | Treatment | RIANIE | 1.00 (0.96, 1.04) |
|  |  | RIANDE | 1.62 (1.56, 1.69) |
| Adjusting for only variables measured at cancer diagnosis, excluding ADG (DAG in Supplemental Figure 1B) | None | RIATCE | 1.69 (1.58, 1.81) |
|  | Stage | RIANIE | 1.09 (1.05, 1.13) |
|  |  | RIANDE | 1.55 (1.48, 1.61) |
|  | Treatment | RIANIE | 1.00 (0.96, 1.04) |
|  |  | RIANDE | 1.69 (1.62, 1.76) |
| Adjusting for age and sex as only confounders (DAG in Supplemental Figure 1C) | None | RIATCE | 1.76 (1.64, 1.88) |
|  | Stage | RIANIE | 1.12 (1.08, 1.16) |
|  |  | RIANDE | 1.57 (1.50, 1.63) |
|  | Treatment | RIANIE | 1.00 (0.96, 1.04) |
|  |  | RIANDE | 1.76 (1.69, 1.83) |
| Binary definitions of mediators with 6-month cutoff for time to treatment initiation | None | RIATCE | 1.62 (1.52, 1.73) |
|  | Stage | RIANIE | 1.08 (1.04, 1.12) |
|  |  | RIANDE | 1.50 (1.44, 1.56) |
|  | Treatment | RIANIE | 1.01 (0.97, 1.05) |
|  |  | RIANDE | 1.61 (1.54, 1.67) |
| Binary definitions of mediators with 3-month cutoff for time to treatment initiation | None | RIATCE | 1.62 (1.52, 1.73) |
|  | Stage | RIANIE | 1.08 (1.04, 1.12) |
|  |  | RIANDE | 1.50 (1.44, 1.56) |
|  | Treatment | RIANIE | 1.00 (0.97, 1.04) |
|  |  | RIANDE | 1.62 (1.55, 1.68) |
| Binary definitions of mediators with 1-month cutoff for time to treatment initiation | None | RIATCE | 1.70 (1.59, 1.81) |
|  | Stage | RIANIE | 1.08 (1.04, 1.12) |
|  |  | RIANDE | 1.57 (1.50, 1.63) |
|  | Treatment | RIANIE | 1.00 (0.96, 1.04) |
|  |  | RIANDE | 1.70 (1.63, 1.77) |
| Ordinal mediators | None | RIATCE |  |
|  | Stage | RIANIE |  |
|  |  | RIANDE |  |
|  | Treatment | RIANIE |  |
|  |  | RIANDE |  |
|  | None | RIATCE |  |
|  | Stage | RIANIE |  |
|  |  | RIANDE |  |
|  | Treatment | RIANIE |  |
|  |  | RIANDE |  |

**Details of mediation analysis using randomized interventional analogues**

##### **Brief overview of effect decomposition using randomized interventional analogues of effects** ^80^

An alternative to the unidentifiable natural indirect effects in scenarios with post-exposure confounding of the M-O relationship are the randomized analogues of interventional effects (RIA), initially described by Didelez et al.^106^ and Geneletti,^107^ then adapted by Vanderweele et al.^80^ for implementation in mediation analyses with post-exposure confounding. An adaptation of this approach has also been demonstrated by Li et al.^82^ in the identification in the mediated effects of deprivation on BC mortality, mediated by stage at diagnosis and time to treatment initiation. To identify analogues of ‘interventional’ effects, mediators are set to a value which is randomly selected from the *distribution* of that mediator, conditioned on either exposure or non-exposure.^80^

Under this framework, the randomized interventional analogue of the total causal effect (RIATCE) of exposure *A* = *a*, relative to non-exposure *A* = *a**, on the outcome *Y,* is defined as:

$$\left( 1 \right) RIATCE= E(Y_{aG_{a}|C}) - E(Y_{a^{*}G_{a^{*}}|C})$$

Whereby, *G_a|C_* denotes a random selection of a value of the mediator from the distribution of the mediator among those with exposure level *a*, conditioning on covariates *C*; and therefore, *Y_aGa|C_* denotes the outcome that would be observed under given exposure *a* and *G_a|C_.* Similarly, *Y_aGa|C_* denotes the outcome that would be observed under given exposure *a** and a random selection of a value of the mediator among those with exposure *a**. Under this framework, the RIATCE can be decomposed into the randomized interventional analogues of natural direct and indirect effects (RIANDE and RIANIE). The RIANIE is defined by equation 2.^80^

The RIANIE contrasts the expected outcome of someone who is exposed (*a*) being randomly assigned a value from the distribution of *M* if they are exposed (*G_a|C_*), and the expected outcome of someone who is exposed (*a*) being randomly assigned a value of *M* from the distribution that *M* would take, given non-exposure (a*).The RIANDE contrasts the expected outcome of someone who is exposed (a) being randomly assigned a value from the distribution of M if they were unexposed (*G_a*|C_*), to the expected value of the outcome of someone who is unexposed (a*) being randomly assigned a value from the distribution of M given non-exposure (*G_a*|C_*), defined by equation 3.

As such, the decomposition of the RIATCE into the RIANIE and RIANDE is expressed by equation 4.

}

Vanderweele et al. stipulate the following assumptions required for the identification of the RIANIE,^80^ RIANDE, and RIATCE in scenarios involving multiple mediators and post-exposure confounding:

1. No unmeasured exposure-outcome confounding, conditional on covariates C, denoted by:
2. No unmeasured mediator outcome confounding, conditional on A, C, and L, denoted by:
3. No unmeasured exposure-mediator confounding, conditional on C, denoted by:

Under these assumptions, the RIANIE and RIANDE can be identified using the following 2 equations:^80^

Whereby, *l* denotes the post-exposure confounder of the mediator-outcome relationship. In the proposed study, the post-exposure confounder when considering the natural indirect effect through time to treatment initiation, is stage at diagnosis.

To contrast the effects of ‘counterfactual’ values of the exposure, mediator, and post-exposure confounder, this approach uses duplications of the observed dataset.^80^ Each duplication corresponds to each ‘counterfactual’ or hypothetical combination of values of exposure (*a*), mediator (*m*), and post-exposure confounder (*l*). The RIANIE and RIANDE can then be identified using the outcome models using the duplicated dataset, with weights calculated using the observed dataset.^80^ Regression equations are then used to predict the following terms: *P*(*a | c*), *P*(*l | a*, c*), and *P*(*m | l, a*, c*).^80^ These terms are then used in the calculation of weights for each observation, according to the following:

To decompose the RIATCE of diagnosis with NAPD on the hazards of mortality following cancer diagnosis, mediated through stage at diagnosis and time to treatment initiation, as illustrated by figure 1, we conducted 3 analyses: (1) the RIATCE of diagnosis with NAPD (*A=*1), relative to not being diagnosed with NAPD (A=0), on the hazards of mortality following cancer diagnosis (2) the RIANIE of diagnosis with NAPD (A=1) on the hazards of mortality following cancer diagnosis, relative to those without NAPD (A=0), mediated by stage at diagnosis; (3) the effect of diagnosis with NAPD (A=1), relative to not being diagnosed with NAPD (A=0), on the hazards of mortality following cancer diagnosis, mediated by time to treatment initiation. Each analysis requires the separate calculations of weights and duplications of the observed dataset.

#### Analysis 1: RIATCE of diagnosis with NAPD on mortality following cancer diagnosis

To compute weights to estimate the RIATCE, a logistic regression model of the odds of having a diagnosis of NAPD was set on our covariates measured at the time of cancer diagnosis. The resulting model was used to predict the propensity of each person having a diagnosis with NAPD, conditional on age at cancer diagnosis, sex assigned at birth, as well as income quintile, rurality of residence, and access to a family physician, at the time of cancer diagnosis. This propensity score was then used in the calculation of the stabilized weights by exposure (diagnosis with NAPD or not diagnosed with NAPD), according to the following weights in equations 7 and 8, respectively:

A Cox proportional model of the hazards of mortality following cancer diagnosis was then fit on diagnosis with NAPD, using the above weights.^80^ The resulting HR and 95% confidence interval associated with NAPD was then estimated the RIATCE of diagnosis with NAPD on the hazards of mortality following cancer diagnosis, adjusting for age at cancer diagnosis, sex assigned at birth, as well as income quintile, rurality of residence, and access to a family physician, at the time of cancer diagnosis. The RIATCE was presented as HR with 95% confidence intervals.

#### Analysis 2: effect of diagnosis with NAPD on mortality following cancer diagnosis, mediated by stage at diagnosis

To compute the weights in this analysis, we used 2 regression models: (1) *P*(*a | c*), logistic regression of having NAPD on age at cancer diagnosis, sex assigned at birth, as well as income quintile, rurality of residence, and access to a family physician, at the time of cancer diagnosis. and *P*(*m | a, c*), an ordinal logistic regression of stage at diagnosis on age at cancer diagnosis, sex assigned at birth, as well as income quintile, rurality of residence, and access to a family physician, at the time of cancer diagnosis and diagnosis with NAPD. The observed dataset was then duplicated with each duplication setting a new variable, a*, representing the counterfactual exposures, 0 or 1. Stabilized weights were calculated using the predicted odds of *a and m,* according to the following equation:

The 3 datasets (1 observed, 2 duplicates), along with their computed weights were then merged. To identify the RIANDE, a Cox proportional hazards model of mortality following cancer diagnosis was then fit on diagnosis with NAPD, using the stabilized weights in equation 9 for all observations with *a** = 0. To identify the RIANIE through stage at diagnosis, a Cox proportional hazards model of mortality following cancer diagnosis was fit on the counterfactual exposure a*, using the stabilized weights from equation 9 for all observations with NAPD (*a* = 1). The RIANIE and RIANDE were presented as HR and 95% confidence intervals.

#### Analysis 3: effect of diagnosis with NAPD on mortality following cancer diagnosis, mediated by time to treatment initiation

As mentioned previously, stage at diagnosis acts as a post-exposure confounder of the pathway between time to treatment initiation and mortality following cancer diagnosis; therefore, the weightings used in the models for analysis 3 incorporated propensity for the distribution of time to treatment initiation. For the calculation of weights in this analysis, we used 3 regression models: (1) *P*(*a | c*), logistic regression of having NAPD on age at cancer diagnosis, sex assigned at birth, as well as income quintile, rurality of residence, and access to a family physician, at the time of cancer diagnosis; (2) *P*(*l | a*, c*), an ordinal logistic regression of stage at diagnosis (I, II,III,IV) on age at cancer diagnosis, sex assigned at birth, as well as income quintile, rurality of residence, and access to a family physician, at the time of cancer diagnosis and diagnosis with NAPD; and (3) *P*(*m | l, a, c*), logistic regression of time to treatment initiation on age at cancer diagnosis, sex assigned at birth, as well as income quintile, rurality of residence, and access to a family physician, at the time of cancer diagnosis, diagnosis with NAPD, and stage at diagnosis. The observed dataset was then duplicated with each duplication setting a new variable, a*, representing the counterfactual exposure, to 0 or 1. Stabilized weights were calculated using the predicted odds of *a, m,* and *l,* according to the following equation:

The 3 datasets (1 observed, 2 duplicates), along with their computed weights were then merged. To identify the RIANDE, a Cox proportional hazards model of mortality following cancer diagnosis was then fit on diagnosis with NAPD, using the stabilized weights in equation 10 for all observations with *a** = 0. To identify the RIANIE through time to treatment initiation, a Cox proportional hazards model of mortality following cancer diagnosis was fit on the counterfactual exposure a*, using the stabilized weights from equation 10 for all observations with NAPD (*a* = 1). The RIANIE and RIANDE were presented as HR and 95% confidence intervals.
